# Single-photon emitters in PECVD-grown silicon nitride films: from material growth to photophysical properties

**DOI:** 10.1515/nanoph-2024-0506

**Published:** 2025-04-29

**Authors:** Zachariah O. Martin, Alexander Senichev, Pranshu Maan, Mustafa G. Ozlu, Miroslava Marinova, Zhongxia Shang, Alexei Lagutchev, Alexandra Boltasseva, Vladimir M. Shalaev

**Affiliations:** Elmore Family School of Electrical and Computer Engineering, Purdue University, West Lafayette, IN, 47907, USA; Purdue Quantum Science and Engineering Institute, Purdue University, West Lafayette, IN, 47907, USA; Birck Nanotechnology Center, Purdue University, West Lafayette, IN, 47907, USA; Quantum Science Center, Department of Energy, A National Quantum Information Science Research Center of the U.S, Oak Ridge National Laboratory, Oak Ridge, TN 37830, USA

**Keywords:** quantum emitters, single-photon emitters, silicon nitride, chemical vapor deposition, quantum photonics

## Abstract

Silicon nitride (SiN) is a key material for quantum photonics due to its wide transparency window, high refractive index, low optical losses, and semiconductor foundry compatibility. We study the formation of single-photon emitters in SiN films grown by plasma-enhanced chemical vapor deposition (PECVD), exploring their photophysical properties and dependence on growth conditions. Emitters were observed across the entire range of nitrogen-to-silicon precursor ratios, from silicon-rich to nitrogen-rich conditions, enabled by the low background fluorescence. We demonstrate single-photon emitters in SiN films with a higher refractive index (1.8–1.9) compared to our previous reports (∼1.7). Notably, nitrogen-rich, thinner films yield particularly bright emitters with shorter emission lifetimes, likely due to more efficient annealing. Silicon-rich SiN films exhibit red-shifted emission, suggesting that composition may provide a mechanism for wavelength tuning. These findings establish the feasibility of emitters formation in foundry standard PECVD tools, advancing the scalability and lab-to-fab transition of SiN-based quantum photonic technologies.

## 1 Introduction

Quantum photonics is one of the leading platforms for implementing quantum computing [1], [2] and communication technologies [3], [4]. Photons offer robustness against decoherence and multiple degrees of freedom for encoding quantum information, while advances in integrated photonics enable scalable on-chip platforms. Solid-state single-photon emitters (SPEs), such as color centers in wide-bandgap materials and semiconductor quantum dots, are essential resources for quantum photonics [5], [6], [7]. Their integration with photonic integrated circuits (PICs) facilitates on-chip generation, manipulation, and detection of quantum states of light, advancing the development of scalable quantum technologies [8].

To achieve scalable PICs with SPEs, on-chip integration within a technologically mature and foundry-friendly platform is essential. Silicon nitride (SiN) has emerged as the leading material for quantum photonic integrated circuits, primarily due to its low-loss in SiN waveguides, broad transparency window, nonlinear properties, and established use in commercial photonics [9], [10], [11], [12]. The integration of quantum light sources with on-chip photonic circuits has been achieved using stoichiometric Si_3_N_4_ waveguides [13], [14]. Additionally, low-autofluorescence nitrogen-rich SiN_x_ has been employed to integrate visible-range quantum emitters with on-chip photonic circuits [15], [16]. Solid-state quantum emitters, such as color centers in diamond (e.g., nitrogen vacancy and silicon vacancy centers), defect centers in two-dimensional hexagonal boron nitride [17], and III-V quantum dots, can potentially emit single photons on demand and have demonstrated high single-photon purity and indistinguishability [5], [18]. These quantum emitters are critical for quantum photonic applications, where the ability to generate and manipulate single photons is essential for advancing quantum communication and computing technologies. However, integrating these SPEs with scalable SiN photonic circuitry platforms has remained a challenge [19], [20], [21], [22]. Although recent advances have been made in the hybrid integration approach – where an SPE in one material is coupled to waveguides made of SiN – this method is typically time-consuming and labor-intensive, limiting its scalability for large-scale quantum photonic systems [20], [21], [22], [23], [24], [25], [26]. As a result, research efforts have increasingly focused on the monolithic integration of color centers with photonic circuitry, which offers a more scalable and efficient solution.

Previously, we demonstrated SPEs in samples having nitrogen-rich SiN films grown on silica (SiO_2_) substrates [27]. These films were produced by high-density plasma chemical vapor deposition (HDPCVD) followed by thermal annealing [28] and exhibited intrinsic quantum emitters with high single-photon purity, brightness, and linear polarization of emission [27]. We earlier explored the optical properties of these SPEs in the HDPCVD SiN/SiO_2_ samples as a function of temperature [29], demonstrated their monolithic integration with photonic circuitry elements [30], and achieved site-controlled fabrication [31].

One limiting factor was that the optimal HDPCVD growth recipe for low-autofluorescence SiN films, required for observing SPEs, led to a reduced refractive index (∼1.7). The lower refractive index is suboptimal for waveguide fabrication as it decreases light confinement, increases propagation losses, and reduces overall efficiency. Low-autofluorescence SiN films with a high refractive index comparable to stoichiometric Si_3_N_4_ can be produced by plasma-enhanced chemical vapor deposition (PECVD) [15], [16]. However, the formation of SPEs in these films had not been studied.

In this work, we investigate the formation of SPEs in SiN films grown by PECVD and subjected to rapid thermal annealing (RTA). Our previous study [27] demonstrated that RTA process creates single-photon emitters in SiN films deposited by HDPCVD on SiO_2_-coated silicon substrates. Here, we extend this approach to PECVD-grown SiN films, aiming to enable emitter formation using foundry-compatible deposition tools while achieving low background fluorescence and a higher refractive index than previously reported.

## 2 Fabrication of low-autofluorescence PECVD SiN

We deposited a series of SiN films by PECVD to explore their properties as a function of nitrogen-to-silicon precursors ratio, focusing on background fluorescence and refractive index. Low background fluorescence is crucial for addressing single-photon emitters, while the refractive index affects light confinement in waveguides and interactions with couplers and resonators. Previous studies have shown that both properties can be tuned in PECVD-grown SiN by adjusting the precursor ratio [15], [16].

We used an Axic Benchmark PECVD system to grow SiN films, varying the gas flow ratio *R* of ammonia (NH_3_) to silane (SiH_4_) from 0.1 to 3.0 to cover Si-rich to N-rich conditions. Films were deposited directly on Si substrates to facilitate ellipsometry data analysis and extraction of the refractive index. Details of PECVD growth parameters, including gas flow rates and NH_3_/SiH_4_ ratios, are provided in the Supplementary Information (Table S1). PECVD SiN/Si samples were fully characterized before and after thermal annealing, including ellipsometry to obtain the refractive index and photoluminescence (PL) imaging and spectroscopy to analyze fluorescence. The samples were annealed using RTA process at 1,100 °C for 120 s under a nitrogen (N_2_) flow, following the same conditions as our previous study on HDPCVD-grown SiN [27].

**Figure 1:**
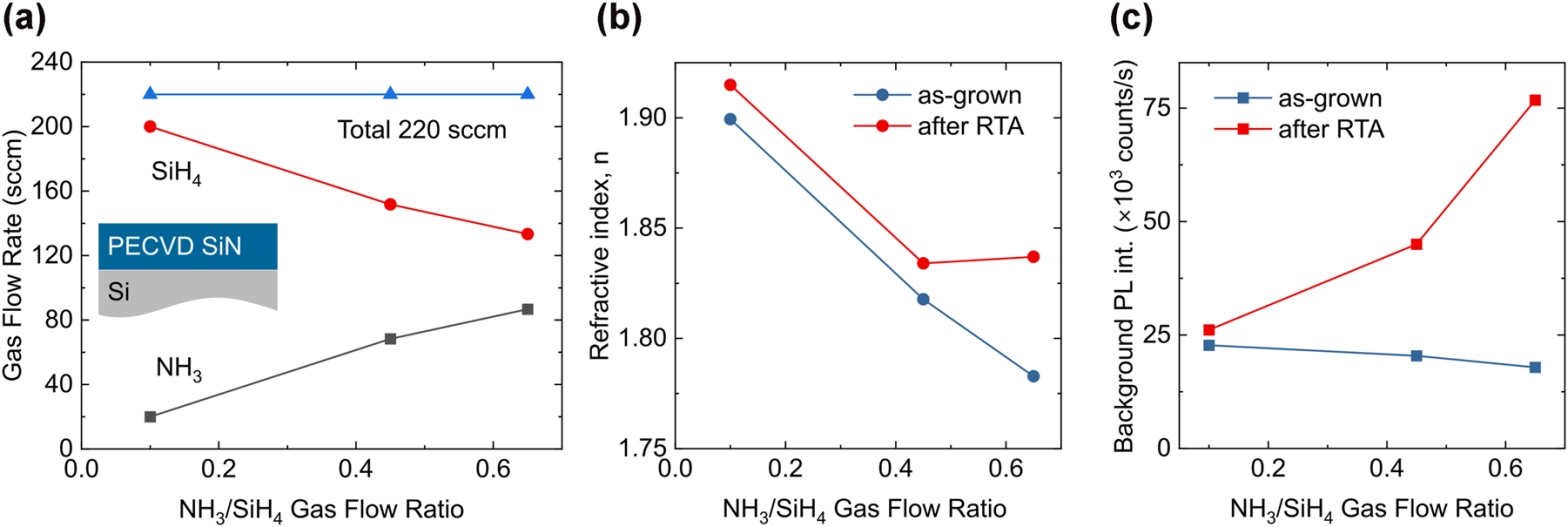
PECVD growth conditions for Si-rich SiN deposition. (a) Gas flow rates of ammonia (NH_3_) and silane (SiH_4_) for NH_3_/SiH_4_ flow ratios *R* ranging from 0.1 to 0.65, with a constant total gas flow of 220 sccm. (b) Refractive index as a function of the NH_3_/SiH_4_ flow ratio, measured before (blue symbols) and after (red symbols) RTA. (c) Background photoluminescence (PL) intensity before (blue symbols) and after (red symbols) RTA, measured using 532 nm laser excitation at 500 μW.

We correlated the refractive index with background fluorescence counts from PL mapping for the PECVD SiN samples grown under different *R*. Figure 1a shows the NH_3_ and SiH_4_ flow rates for Si-rich samples and the corresponding values for *R* at a total gas flow of 220 sccm. The refractive index of the resulting films at wavelength of 600 nm was determined by fitting ellipsometry data with the Cody-Lorentz model. As shown in Figure 1b, the refractive index decreases with increasing *R* in as-grown films. Background fluorescence remains largely unchanged in as-grown samples but increases after RTA (Figure 1c), with a more pronounced effect at higher *R*. This trend correlates with a notable increase in the refractive index after annealing for the sample grown at *R* = 0.65.

**Figure 2:**
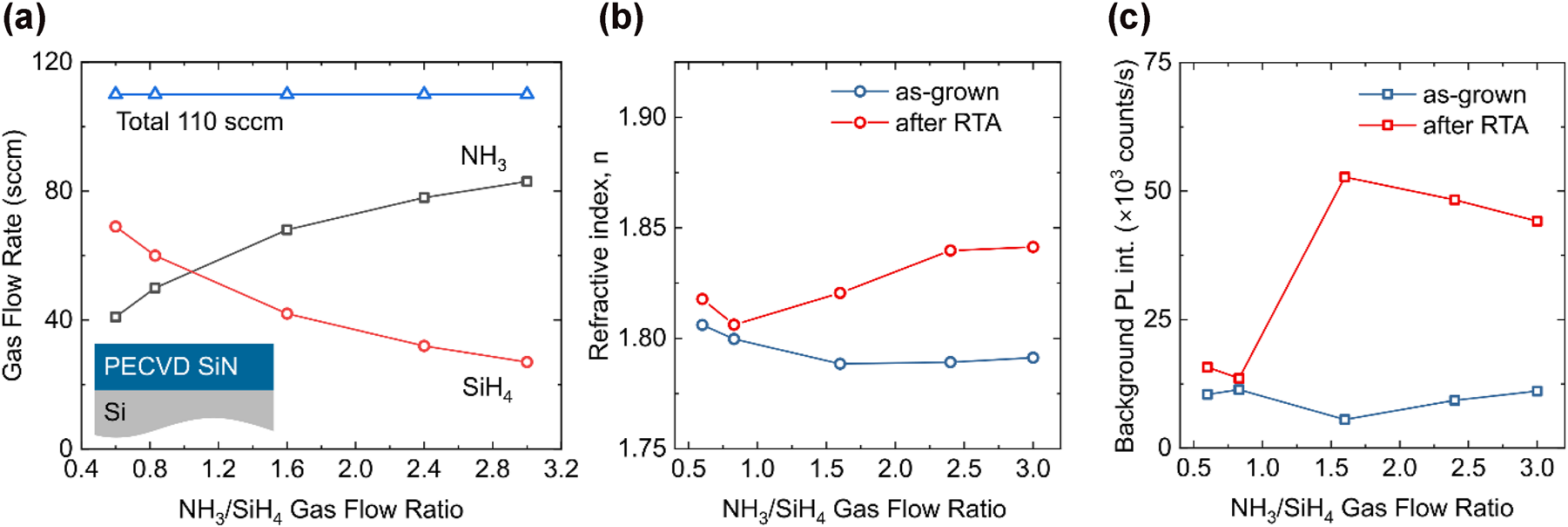
PECVD growth conditions for N-rich SiN deposition. (a) Gas flow of ammonia (NH_3_) and silane (SiH_4_) used to achieve NH_3_/SiH_4_ flow ration from 0.6 to 3.0. The total gas flow was kept constant at 110 sccm. (b) Refractive index as a function of ammonia to silane flow ratio measured before (blue symbols) and after (red symbols) RTA. (c) Background PL intensity before (blue symbols) and after (red symbols) RTA measured using 532 nm laser excitation at 500 μW.

A similar trend was observed for N-rich samples, which were grown with a lower total gas flow of 110 sccm due to system limitations on NH_3_ flow (Figure 2). In as-grown N-rich films, the refractive index *n* decreases slightly with increasing *R* and saturates just below 1.8, while background fluorescence remains low. After annealing, both the refractive index and background fluorescence increase with *R*. However, this effect becomes particularly pronounced for *R* > 0.83 under these growth conditions.

The changes observed before and after RTA can be attributed to hydrogen incorporation during PECVD growth [32]. The hydrogen incorporation increases with the increase of the NH_3_ flow further lowering the refractive index. Upon annealing, the release of weakly bonded hydrogen results in material densification and an increase in the refractive index [32], [28]. This is further supported by the reduction in SiN film thickness after annealing (Supplementary Information, Fig. S1). The rise in background fluorescence may result from reduced non-radiative recombination pathways after thermal annealing. Despite differences in total gas flow and growth rate between the sample sets, the increase in refractive index and background fluorescence follows the same trend and becomes more pronounced at higher *R*.

The refractive index trends for both sets of samples (Figures 1b and 2b) are consistent with previous reports, showing a decrease with increasing *R* for as-grown samples [15], [16]. Despite minor variations before and after RTA, background fluorescence remains significantly lower than reported in the literature and in our previous samples across the entire *R* range, including near-stoichiometric and silicon-rich growth conditions, which is critical for observation of single-photon emitters.

## 3 Characterization of single-photon emitters in PECVD SiN

We then studied the formation and properties of SPEs in PECVD-grown SiN films. In addition to PECVD SiN/Si samples, we prepared samples grown on SiO_2_-coated silicon substrates using the growth parameters shown in Figures 1a and 2a (Supplementary Information, Table S1). This substrate choice allows direct comparison of PECVD-grown SiN/SiO_2_ samples with those from our previous study, where single-photon emitters were observed in HDPCVD-grown SiN films under nitrogen-rich conditions. In that study, emitter formation was only observed in SiN films grown on SiO_2_ layers [27]. Similar to the SiN films grown directly on Si substrates, PECVD SiN/SiO_2_ samples were annealed under the same RTA conditions at 1,100 °C in a nitrogen atmosphere for 2 min, consistent with our previous study [27].

Confocal PL intensity maps reveal isolated emitters in both sample series: those grown directly on silicon substrates (SiN/Si) and on SiO_2_-coated silicon substrates (SiN/SiO_2_). In the main text, we focus on PECVD-grown SiN/SiO_2_ samples and compare the photophysics of single-photon emitters with previous results from HDPCVD-grown SiN/SiO_2_. The Supplementary Information details PECVD SiN/Si samples for comparative analysis, as their behavior is similar to PECVD SiN/SiO_2_ (Supplementary Information, Figs. S2–S4).

**Figure 3:**
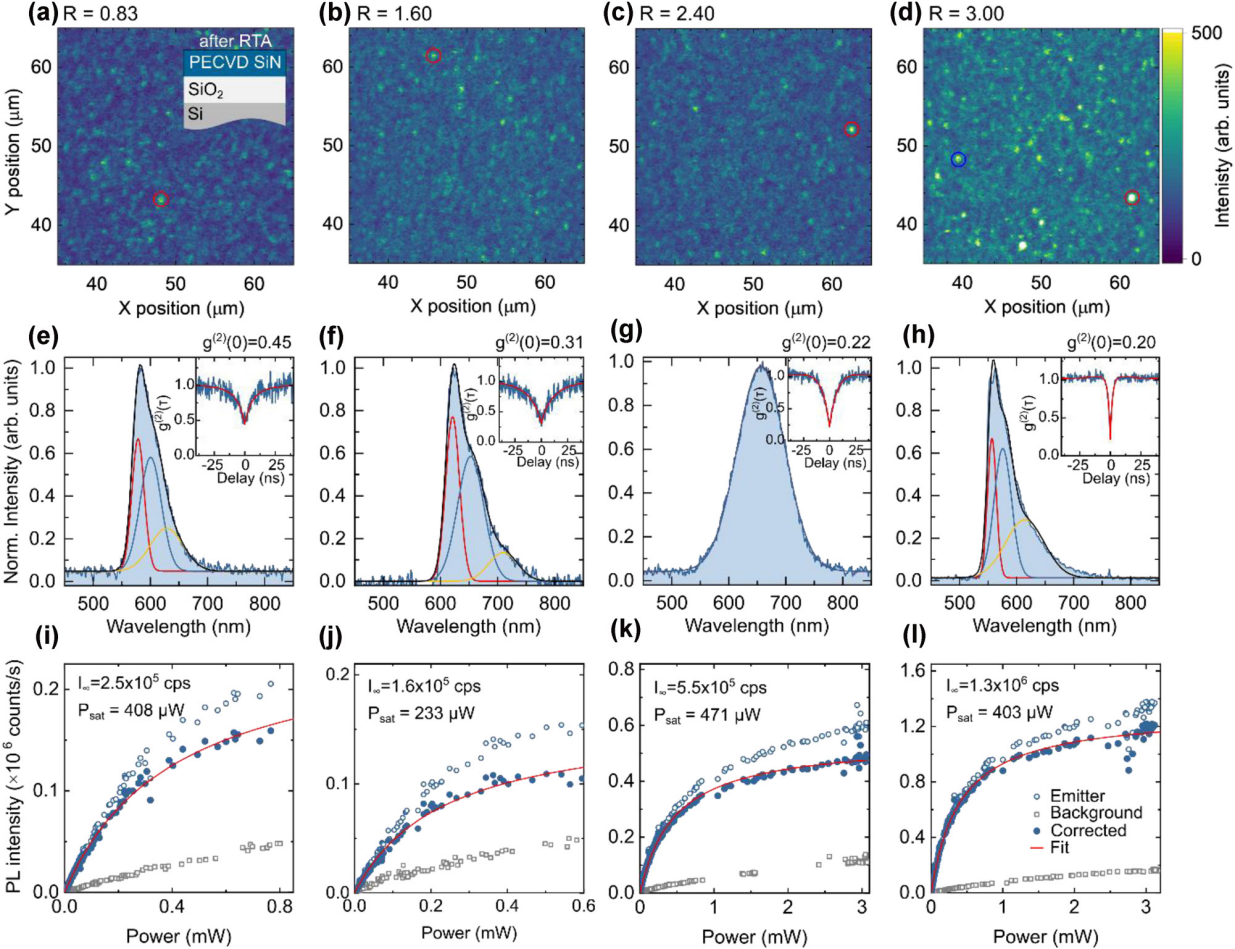
Characterization of single-photon emitters in PECVD-grown SiN/SiO_2_ samples. Samples were fabricated with an NH_3_ to SiH_4_ gas flow ratio ranging from 0.83 to 3.0. (a–d) Confocal PL intensity maps (30 × 30 μm^2^) after thermal annealing, showing isolated emitters. (e–h) PL spectra of the corresponding emitters highlighted in (a–d), measured under 532 nm laser excitation at 500 μW. All PL spectra are background-corrected. Insets: Second-order autocorrelation histograms g^(2)^(τ), confirming single-photon emission characteristics. (i–l) PL intensity as a function of excitation power of the corresponding emitters in (a–d), illustrating the saturation behavior typical of single-photon emitters. Full blue circles represent background-corrected saturation behavior, fitted with $I\left(P\right)=I_{\infty }\times P/\left(P+P_{\mathrm{sat}}\right)$ .

We begin with PECVD SiN films grown under nitrogen-rich conditions shown in Figure 2a. Details on the PL characterization setup used to probe photophysical properties of emitters, including single-photon purity, emission stability, emission wavelength, and brightness are provided in the Supplementary Information (Section S2). Confocal PL intensity maps of PECVD SiN/SiO_2_ samples after RTA reveal isolated emitters on a low-intensity background (Figure 3a–d). Some emitters were photobleached when excited with a 532 nm laser at 500 μW power. However, we obtained PL spectra, second-order autocorrelation histograms, and saturated intensity curves for emitters from each sample. The characterized emitters are highlighted with red circles in Figure 3a–d.

The PL spectra of the corresponding emitters are shown in Figure 3e–h. As expected for solid-state emitters at room temperature, their spectra are broad. Some emitters exhibit spectra that can be fitted with up to three Gaussian components, similar to those in HDPCVD-grown SiN [27]. The most intense peaks appear between 550 and 650 nm. Further statistical analysis is needed to determine whether PL peaks consistently cluster around specific wavelengths, as previously observed in HDPCVD-grown SiN [27].

The single-photon purity was assessed by measuring the second-order autocorrelation histogram using a Hanbury Brown and Twiss (HBT) setup. Fitting the second-order autocorrelation function yielded g^(2)^(0) values clearly below 0.5, confirming the emitters as single-photon sources (Figure 3e–h **, insets**).

PL intensity measurements as a function of excitation power provide the corresponding saturated intensity of emitters (Figure 3i–l). For samples grown at NH_3_/SiH_4_ ratios from 0.83 to 2.40, the brightest emitters selected per each PL map exhibit saturated intensities on the order of 10^5^ counts per second. In contrast, for the sample grown at *R* = 3.0, we identified particularly bright emitters with saturated intensities on the order of 10^6^ counts per second (Figure 3l), which is on par with emitters in HDPCVD-grown SiN. In the same sample, the less bright emitter indicated with the blue circle shows the same characteristics as the emitters in the samples grown at lower NH_3_/SiH_4_ ratios (Supplementary Information, Fig. S5).

Moreover, this bright emitter shown in Figure 3d exhibits a narrower g^(2)^(0) histogram (Figure 3h **, inset**) with a characteristic τ_1_ time of 1.7 ns, compared to 5–13 ns for emitters in Figure 3e–g, indicating a shorter emission lifetime. This suggests that PECVD-grown SiN can support emitters with higher brightness, shorter emission lifetimes, and potentially greater stability. The increased brightness and faster spontaneous emission rates may result from the influence of material composition and more effective thermal annealing on SPE formation, as films grown at higher *R* are thinner due to reduced SiH_4_ gas flow (Supplementary Information, Fig. S1). This finding provides a potential pathway for optimizing single-photon emitters in PECVD-grown SiN.

**Figure 4:**
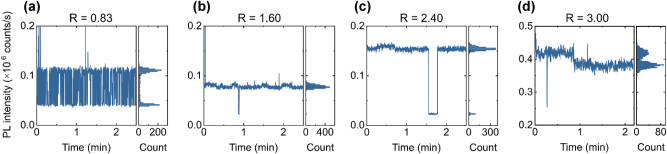
Emission intensity time traces of single-photon emitters from Figure 3. Measurements were performed at an excitation power of 500 μW with a sampling time of 100 ms over 150 s. Left panels: Corresponding histograms of photon emission intensity distributions, illustrating transitions between high- and low-intensity states for some emitters.

The emission time-traces of the corresponding emitters show that some emitters exhibit blinking, switching between high- and low-intensity states (Figure 4). In some cases, switching occurs rapidly between two states (Figure 4a), while in others, it happens only once within a 2-min time window (Figure 4c). The available data does not yet reveal a clear trend in emission stability as a function of the NH_3_/SiH_4_ ratio, though emitters in the samples grown at the lower ratio *R* are more susceptible to photobleaching.

For PECVD SiN/Si samples with SiN films grown directly on silicon substrates, emitters exhibit similar properties. Emitters were observed after RTA across the entire NH_3_/SiH_4_ ratio range from 0.1 to 3.0 (Supplementary Information, Figs. S2–S4). Figure S2 shows PL intensity maps for N-rich samples before and after annealing, demonstrating the appearance of quantum emitters after RTA. Figure S3 presents the photophysical properties of randomly selected photostable emitters from each PL map.

The main difference compared to PECVD SiN/SiO_2_ samples is that emitters in SiN/Si samples are substantially less bright, with saturated intensities ranging from 10^4^ to 10^5^ counts per second. Most likely, losses into high-index Si substrates reduce photon collection efficiency, whereas the lower-index SiO_2_ layer enhances confinement and light extraction. As a result, despite low background fluorescence in PECVD SiN/Si samples, the single-photon purity, characterized by g^(2)^(0) at zero delay, is not always below 0.5, as the emission intensity is comparable to the background PL. However, emitters from both PECVD SiN/Si and SiN/SiO_2_ samples exhibit similar spectral characteristics.

**Figure 5:**
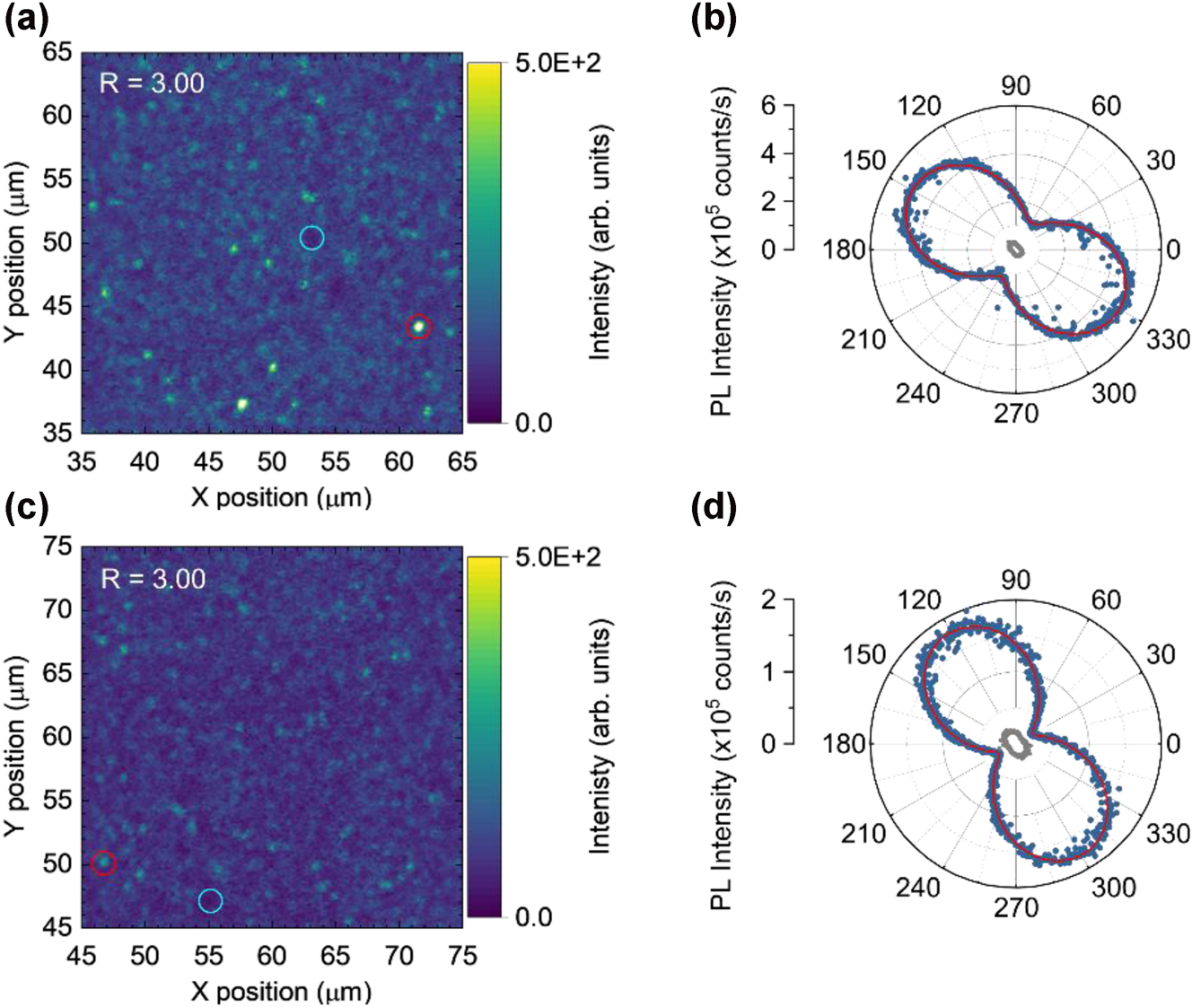
Emission polarization of two emitters from a sample grown at NH_3_/SiH_4_ = 3. (a,c) Confocal PL intensity maps (30 × 30 μm^2^), with single-photon emitters marked by red circles and background polarization measurement positions marked by light blue circles. (b,d) Emission polarization diagrams of the corresponding emitters from (a,c). The emission was not background-corrected. The solid red lines were obtained using a cos^2^ *θ*-fit function applied to the data. Grey data points: background emission polarization measurements. The slight polarization of background PL is likely due to optical elements in the emission collection path, particularly the dichroic mirror used to separate excitation and detection paths.

Next, we characterized the emission polarization of selected single-photon emitters in PECVD SiN/SiO_2_ sample grown at *R* = 3. Figure 5 shows the emission polarization diagrams for two emitters marked in corresponding PL intensity maps. The measured emission exhibits linear polarization, as expected for single-photon emitters. The polarization diagrams (Figure 5b and d) were fitted using a cos^2^ *θ*-form function, yielding visibility values of 0.59 and 0.74 for each emitter, respectively. Background fluorescence polarization measurements (grey data points) were taken from regions marked with light blue circles in the PL maps, indicating slight polarization likely due to optical elements in the detection path, particularly the dichroic mirror. However, the background emission is negligible compared to the emitter signal.

**Figure 6:**
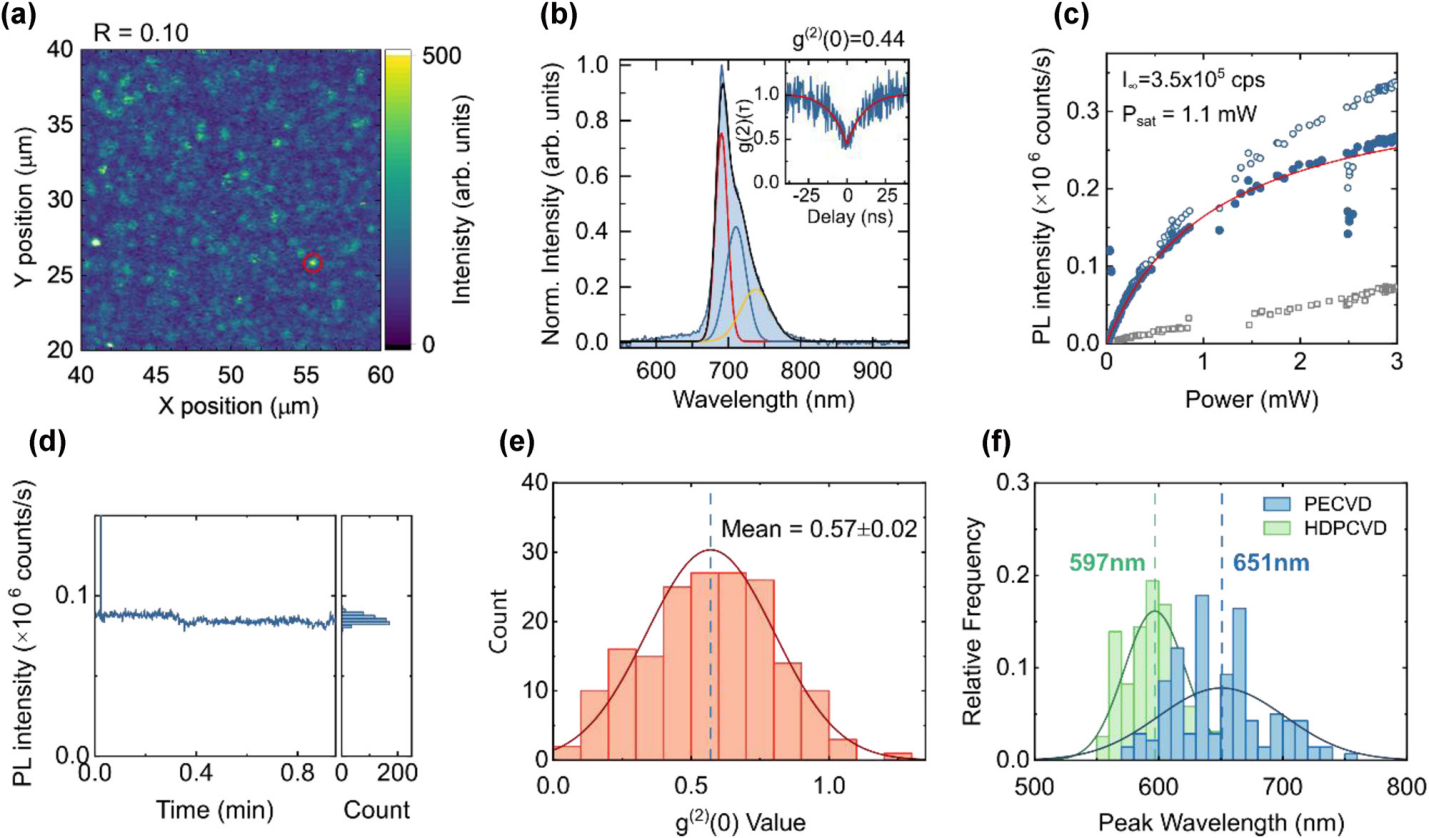
Photophysical properties of single-photon emitters in Si-rich 200-nm-thick PECVD SiN film grown on 100-nm-thick HDPCVD silicon oxide layer. (a) Confocal PL intensity map (20 × 20 μm^2^) after thermal annealing, showing isolated emitters. (b) PL spectrum of the corresponding emitter highlighted in (a), measured under 532 nm laser excitation at 1.5 mW. PL spectrum is background-corrected. Inset: Second-order autocorrelation histogram g^(2)^(τ). (c) PL intensity as a function of excitation power. Full blue circles represent background-corrected saturation behavior, fitted with $I\left(P\right)=I_{\infty }\times P/\left(P+P_{\mathrm{sat}}\right)$ . (d) Emission intensity time-trace. Measurement was performed at an excitation power of 1.5 mW with a sampling time of 100 ms over 60 s. Left panel: Corresponding histogram of photon emission intensity distributions. (e) g^(2)^(0) statistics show modest single photon purity, with the mean g^(2)^(0) value of 0.57±0.02, indicating that almost half of the measured bright spots are single-photon emitters. (f) Comparison of the spectral peak positions of PECVD SiN/SiO_2_ single-photon emitters (light blue) versus the peak positions of HDPCVD SiN/SiO_2_ emitters (light green).

Due to the low background fluorescence observed in PECVD-grown SiN films across all NH_3_/SiH_4_ gas flow ratios, both before and after annealing, single-photon emitters can be identified even in silicon-rich films. These SiN films have a higher refractive index, which is advantageous for photonic structures. Here, we characterize a SiN film grown at the lowest NH_3_/SiH_4_ ratio of 0.1 (Figure 6). This Si-rich SiN film exhibits the highest refractive index after RTA of 1.91 among studied samples and a background fluorescence level of ∼1.3 × 10^4^ counts per second at 500 μW excitation power at 532 nm.

The PL intensity map of the Si-rich PECVD SiN/SiO_2_ sample (Figure 6a) reveals isolated bright spots on a uniform, low-intensity background fluorescence. The photophysical properties of a representative emitter (marked with a red circle) are shown in Figure 6b–d. The second-order autocorrelation function confirms its single-photon nature with a g^(2)^(0) value of 0.44. The emitter exhibits the saturated intensity of 3.5 × 10^5^ counts per second, similar to those observed in PECVD SiN films grown under N-rich conditions. The emission intensity time-trace (Figure 6d) shows stable emission without blinking within the measured ∼60-s window. However, this emitter exhibits a red-shifted PL spectrum (Figure 6b), peaking near 700 nm, in contrast to the 550–650 nm emission range observed for N-rich PECVD samples (Figure 3e–h) and the ∼600 nm most intense peak typically seen in N-rich HDPCVD-grown SPEs at room temperature [27].

To better understand the room-temperature PL properties of emitters in this sample, we characterized 176 emitters and analyzed their antibunching and spectral properties using automated scanning measurements (Fig. 6e and f). The average g^(2)^(0) value at zero delay time is 0.57 ± 0.02 (Figure 6e), which is worse than that previously observed for HDPCVD SiN/SiO_2_ SPEs [27]. This may be due to the lower average brightness of PECVD SiN emitters, while the switching between high- and low-intensity states, may further contribute to reduced single-photon purity.

Next, we analyzed the distribution of PL peak positions. Background-corrected PL spectra show an average peak wavelength of 651 nm for PECVD SiN/SiO_2_ SPEs (Figure 6f **, blue**). This average peak position lies at the longer-wavelength end of the emission range observed in N-rich PECVD SiN samples in this study. For comparison with our previous study, we also grew a control N-rich HDPCVD SiN/SiO_2_ sample (100 nm SiN, 20 nm SiO_2_) and annealed it at 900 °C in a Thermo Scientific Lindberg/Blue M furnace. The emitters in this control sample showed photophysical properties consistent with previously reported HDPCVD SiN/SiO_2_ SPEs after RTA, with an average peak position at 597 nm (Figure 6f **, green**). This confirms that PECVD-grown Si-rich SiN/SiO_2_ SPEs exhibit red-shifted emission compared to N-rich SiN/SiO_2_ and HDPCVD-grown N-rich SiN/SiO_2_ SPEs. Additionally, the peak position distribution for PECVD emitters (Figure 6f **, blue**) is broader than that of HDPCVD emitters, suggesting greater spectral variability. The redshift may result from differences in strain fields or film composition. It may also indicate a distinct emitter origin in PECVD-grown films, such as silicon crystalline nanoparticle formation, which has been reported in Si-rich SiN samples after thermal annealing [33]. We will discuss possible explanations for the variation in spectral properties in more detail in the next section.

## 4 Discussion

In our previous study, we found that SPEs can be formed in HDPCVD-grown SiN/SiO_2_ samples after RTA [27]. Here, we extended this research to PECVD-grown SiN, demonstrating emitter formation with foundry-compatible deposition while maintaining low background fluorescence and achieving a higher refractive index.

The refractive index of PECVD-grown SiN films decreases with increasing nitrogen-to-silicon precursors ratio *R*, consistent with trends reported in the literature [16], [28], [34], [35], [36]. However, our samples exhibit lower-than-expected refractive indices, particularly in Si-rich films. Transmission electron microscopy (TEM) analyses with energy-dispersive X-ray spectroscopy (EDS) confirms a higher silicon content than the stoichiometric Si_3_N_4_ ratio in SiN/Si and SiN/SiO_2_ samples grown at *R* = 0.1 (Supplementary Information, Fig. S6 and Fig. S7), yet the measured refractive index of the as-grown film is 1.9, whereas a value above 2.0 would be expected for such compositions [37]. We assign this observation to hydrogen incorporation from precursors, as hydrogen introduces N–H bonds and increases film porosity, reducing the refractive index. Thermal annealing releases hydrogen, leading to densification and a refractive index increase [32], [28], as observed in our post-annealing ellipsometry measurements. This densification is further supported by the observed reduction in SiN film thickness after annealing (Supplementary Information, Fig. S1).

The increase in refractive index after RTA is more pronounced in samples grown at higher *R*, likely due to greater hydrogen incorporation and more efficient annealing in thinner films, resulting from reduced silane flow. Some samples may not have reached the threshold for complete hydrogen removal, leading to a lower-than-expected refractive index even after annealing. Additionally, variations in RF power, deposition temperature, and growth rate can influence the refractive index for a given *R* ratio, explaining discrepancies with literature values [28], [34]. Nonetheless, the refractive index of PECVD SiN films with SPEs in this study (1.8–1.9) is higher than that of HDPCVD-grown samples in our previous work (∼1.7) [27].

Importantly, the background fluorescence remains low across all NH_3_/SiH_4_ ratios, with only a slight increase post-annealing, possibly due to reduced non-radiative recombination pathways. The background intensity change after RTA correlates with the rise in refractive index. While further studies are needed to clarify this mechanism, the consistently low background fluorescence across different deposition conditions enables clear observation and characterization of single-photon emitters in PECVD SiN films of different composition.

SPEs were observed in samples grown at all NH_3_/SiH_4_ ratios within this study, with comparable brightness, single-photon purity, and stability. This suggests that emitter formation is not strongly dependent on material composition within the studied range. However, notable differences emerge in the most nitrogen-rich sample (PECVD SiN/SiO_2_), where particularly bright emitters (>10^6^ counts per second) with shorter emission lifetimes, estimated from g^(2)^(τ) fitting, are observed. This could be attributed to more efficient RTA in thinner films, as this sample had the lowest SiN thickness among all studied conditions. More effective annealing may lead to improved defect activation or stabilization, favoring the formation of brighter and potentially more stable emitters in PECVD SiN.

The impact of annealing efficiency is further supported by the observation of brighter emitters, reaching intensities above 10^6^ counts per second, in Si-rich films subjected to prolonged thermal annealing in a conventional tube furnace (Supplementary Information, Fig. S8). The process involved slow annealing in a horizontal furnace (ProTemp Products) at 1,000 °C under Ar for 60 min, followed by an additional 60 min at 1,100 °C under N_2_. While this comparison involves a different sample grown directly on a silicon substrate (PECVD SiN/Si), it highlights the potential role of extended annealing in improving emitter properties.

The spectral differences between PECVD SiN/SiO_2_ samples grown under Si-rich and N-rich conditions, as well as in comparison to N-rich HDPCVD-grown SiN, suggest that composition, strain, or even fundamentally different defect structures may influence emitter characteristics. One possible explanation is that the emitters experience different strain environments due to variations in material composition and optical properties between the two films. Strain tuning has been shown to shift the electronic energy levels of solid-state quantum emitters, including quantum dots [38], color centers in diamond [39], [40], and hBN [41], [42]. However, typical strain-induced shifts in SPE energy levels range from 0.5 meV to 5.0 meV, which is insufficient to explain the 170 meV difference in the mean peak positions between HDPCVD SiN/SiO_2_ and PECVD SiN/SiO_2_ emitters.

Another possibility is that the emitters in Si-rich PECVD SiN/SiO_2_ have a different origin than those in N-rich samples deposited by either PECVD or HDPCVD. Previous studies have shown that silicon nanocrystals can form in Si-rich SiN, leading to PL spectral shifts due to quantum confinement effects [33], [43], [44]. The spectral position of PL from samples with these nanocrystals aligns well with our results. However, preliminary TEM/EDS analysis did not reveal Si nanocrystals in our samples (Supplementary Information, Fig. S6 and Fig. S7). Moreover, the emission lifetimes previously reported for Si quantum dots in SiN are on the order of microseconds [33], whereas in our work, the lifetimes estimated from g^(2)^(τ) function fitting are on the order of a few nanoseconds. Other studies suggest the presence of midgap defect centers as potential sources of the observed PL emission [45]. Understanding the role of composition in PL spectral variations could be valuable for tuning SPE emission properties.

Regarding the nature of emitters, the observation of SPEs with similar properties in PECVD SiN films grown on both Si substrates and SiO_2_-coated Si substrates suggests that emitter formation is not strictly dependent on a thick oxide buffer layer. However, since even bare silicon substrates have a thin native oxide layer, the role of the interface in emitter formation remains unclear. One possible mechanism involves hydrogen release during annealing, leading to material densification and atomic bond redistribution. Hydrogen incorporation is well known in PECVD SiN films, and its release during thermal annealing may induce structural reconfiguration, potentially facilitating the formation of defect centers responsible for single-photon emission. Understanding the nature of single-photon emitters in SiN remains an ongoing research effort, and further insights into their formation mechanisms and defect origins will be reported elsewhere.

## 5 Conclusions

This work investigates SPEs formation in PECVD-grown SiN films across a range of NH_3_/SiH_4_ gas flow ratios, comparing samples grown on both Si and SiO_2_-coated Si substrates. Emitters were observed in all studied conditions, with mostly comparable brightness, single-photon purity, and stability. However, particularly bright emitters with shorter emission lifetimes were found in nitrogen-rich, thinner films, likely due to more efficient annealing. The refractive index trends and post-annealing changes both suggest hydrogen incorporation and subsequent release, influencing material densification and possibly emitter activation. The spectral redshift of SPEs in Si-rich films compared to N-rich films and HDPCVD-grown SiN suggests compositional or strain-related effects, which can be used for emission tunability. The consistently low background fluorescence across deposition conditions enables clear SPE observation, demonstrating the potential for integrating PECVD-grown SiN SPEs into scalable photonic platforms. These findings highlight the feasibility of emitter formation in foundry-standard PECVD tools, which is critical for the scalability and lab-to-fab transition of SiN-based quantum photonic technologies.

## Supplementary Material

Supplementary Material Details

## Supplementary Information

See the Supplementary Information for additional information.
