# Supplementary material for: Single-photon emitters in PECVD-grown silicon nitride films: from material growth to photophysical properties
Source: Nanophotonics. 2025 Apr 29;14(11):1783–93. doi: 10.1515/nanoph-2024-0506 (PMC12133249; doi:10.1515/nanoph-2024-0506)
Supplement: Supplementary file 1 — Supplementary Material Details [file j_nanoph-2024-0506_suppl_001.pdf]

Supplementary Information

Single-Photon Emitters in PECVD-Grown Silicon Nitride Films: From Material Growth to Photophysical Properties

Zachariah O. Martin[1,2,#], Alexander Senichev[1,2,#], Pranshu Maan[1,2], Mustafa G. Ozlu[1], Miroslava Marinova[1], Zhongxia Shang[3], Alexei Lagutchev[1], Alexandra Boltasseva[1,2], Vladimir M. Shalaev[1,2]*

[1]Elmore Family School of Electrical and Computer Engineering, Birck Nanotechnology Center and Purdue Quantum Science and Engineering Institute, Purdue University, 610 Purdue Mall, West Lafayette, IN, 47907, USA

[2]Quantum Science Center, Department of Energy, A National Quantum Information Science Research Center of the U.S, Oak Ridge National Laboratory, 1 Bethel Valley Road, Oak Ridge, TN 37830, USA

[3]Birck Nanotechnology Center, Purdue University, 1205 Mitch Daniels Blvd, West Lafayette, IN 47907, USA

[#]These authors contributed equally to this work.

*Corresponding author: shalaev@purdue.edu

Number of pages: 8

Number of figures: 8

Number of tables: 1

Section S1. PECVD growth parameters

The samples were grown using an Axic Benchmark PECVD system. For silicon nitride (SiN) film deposition, the gas flow ratio *R* of ammonia (NH_3) to silane (SiH_4) was varied from 0.1 to 3.0, covering Si-rich to N-rich conditions. The specific flow rates of NH_3 and SiH_4 for each ratio and total gas flow rates are listed in **Table S1**. Note that in order to keep the gas fluxes within the tool parameters, the N-rich samples (D-H) were grown with the total gas flow rate halved (110 sccm). The maximum ammonia flow rate available in our PECVD system is 100 sccm. The chamber pressure was set to 600 mTorr, RF power to 150 W, and temperature to 300°C. All samples were grown for 5 minutes at the total flow of 220 sccm (10 minutes at 110 sccm). The SiN samples were annealed using rapid thermal annealing (RTA) processor at 1100°C for 120 seconds in a nitrogen (N_2) atmosphere with a Jipelec Jetfirst RTA system.

Growth	Sample	Ratio (NH_3/SiH_4)	NH_3 Flow (sccm)	SiH_4 Flow (sccm)	Total Flow (sccm)
**Si-rich**	A	0.1	20	200	220
	B	0.45	68.3	151.7	
	C	0.65	86.7	133.3	
**N-rich**	D	0.6	41	69	110
	E	0.83	50	60	
	F	1.6	68	42	
	G	2.4	78	32	
	H	3 .0	83	27	

Table S1. Growth parameters for PECVD-SiN films including gas flow rates, total flow, and NH_3/SiH_4 ratios.

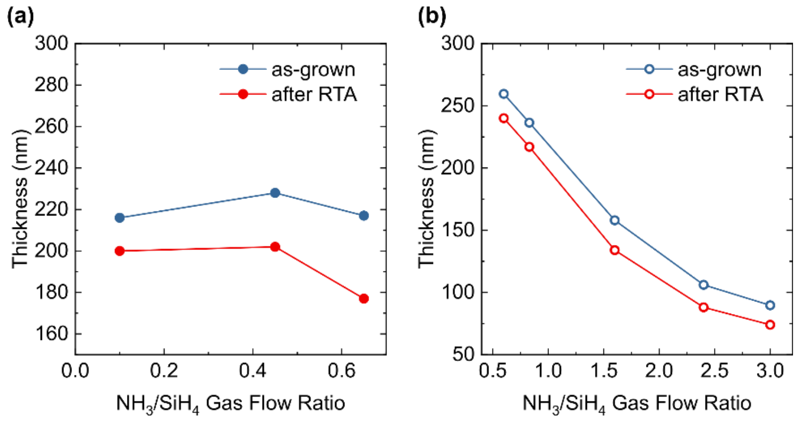

Figure S1. Thickness of PECVD SiN films grown under (a) Si-rich and (b) N-rich growth conditions measured before (blue symbols) and after (red symbols) rapid thermal annealing (RTA).

Section S2. Photoluminescence characterization setup

We use a custom-built scanning confocal microscope with a 100 μm pinhole based on a commercial inverted microscope body (Nikon Ti−U) to perform room temperature optical characterization of the films after growth. A P-561 piezo stage driven by an E-712 controller (Physik Instrumente) scanned a 100x air objective with a 0.95 numerical aperture (NA) to measure PL intensity maps. A continuous wave 532 nm laser (RGB Photonics) was used for excitation. The excitation beam was reflected off a 550 nm long-pass dichroic mirror (DMLP550L, Thorlabs), and a 532 nm edge-pass filter and a 550 nm long-pass filter (FEL0550, Thorlabs) removed the residual pump beam. Single-photon emission was recorded using a single-photon counting module (SPCM-AQRH, Excelitas) with 69% photon detection efficiency at 650 nm. Autocorrelation measurements were performed with two avalanche detectors with a 30 ps time resolution and 35% quantum efficiency at 650 nm (PDM, Micro-Photon Devices). Time-correlated photon counting was performed by a correlation card "start-stop" acquisition card with a 4 ps internal jitter (SPC-150, Becker & Hickl). PL spectra were measured using an Ocean Optics QEPro6500 UV-VIS spectrometer.

Section S3. Characterization of single-photon emitters

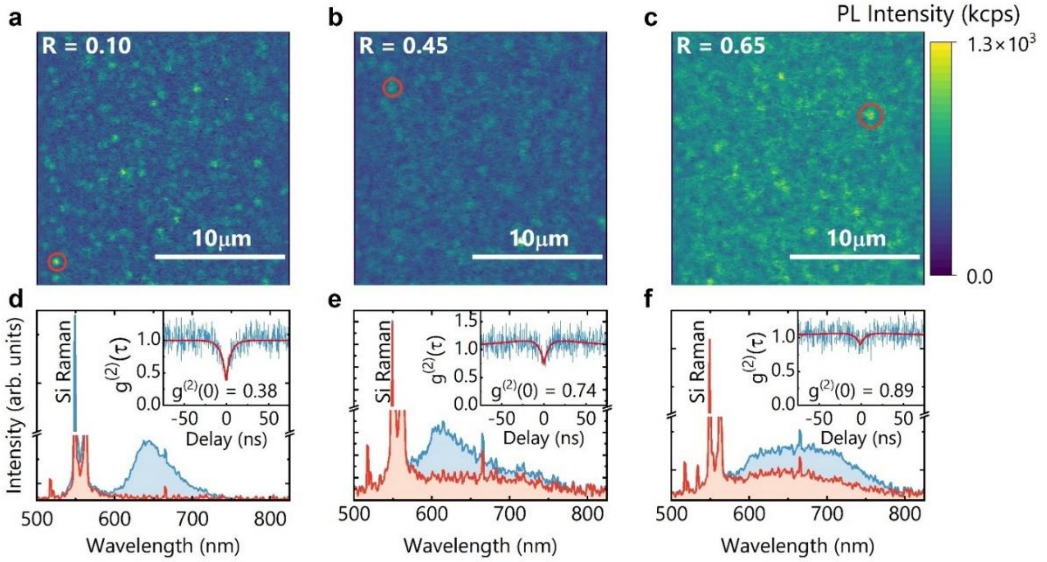

Figure S2. Photophysical properties of SPEs in Si-rich PECVD SiN/Si samples after RTA. (a,b,c) Confocal PL intensity maps of the SiN samples reveal isolated bright spots. NH_3 to SiH_4 flow ratio *R* is indicated in the top left. (d,e,f) PL spectra from the emitter circled in part (a,b,c) as well as the background. Si Raman lines originating from the substrate appear both in emitter and background spectra. Insets: $g^{(2)}(\tau)$ measurements recorded from the selected emitter all show at least some degree of antibunching.

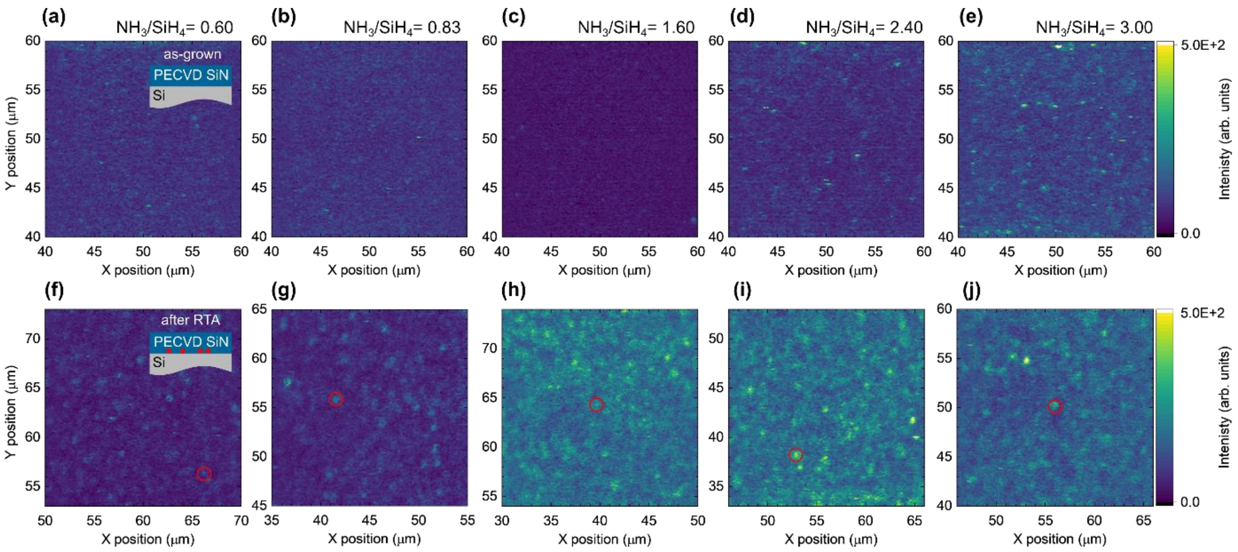

Figure S3. Confocal PL intensity maps (20 × 20 μm²) of N-rich PECVD-grown SiN/Si samples measured (a-e) before and (f-j) after RTA. No single-photon emitters are observed in the as-grown SiN films. Bright speckles in (d) and (e) correspond to fluorescing surface particles that photobleach after a single laser pass. In contrast, single-photon emitters are present in all SiN films after RTA. Representative emitters are highlighted with red circles in (f–j).

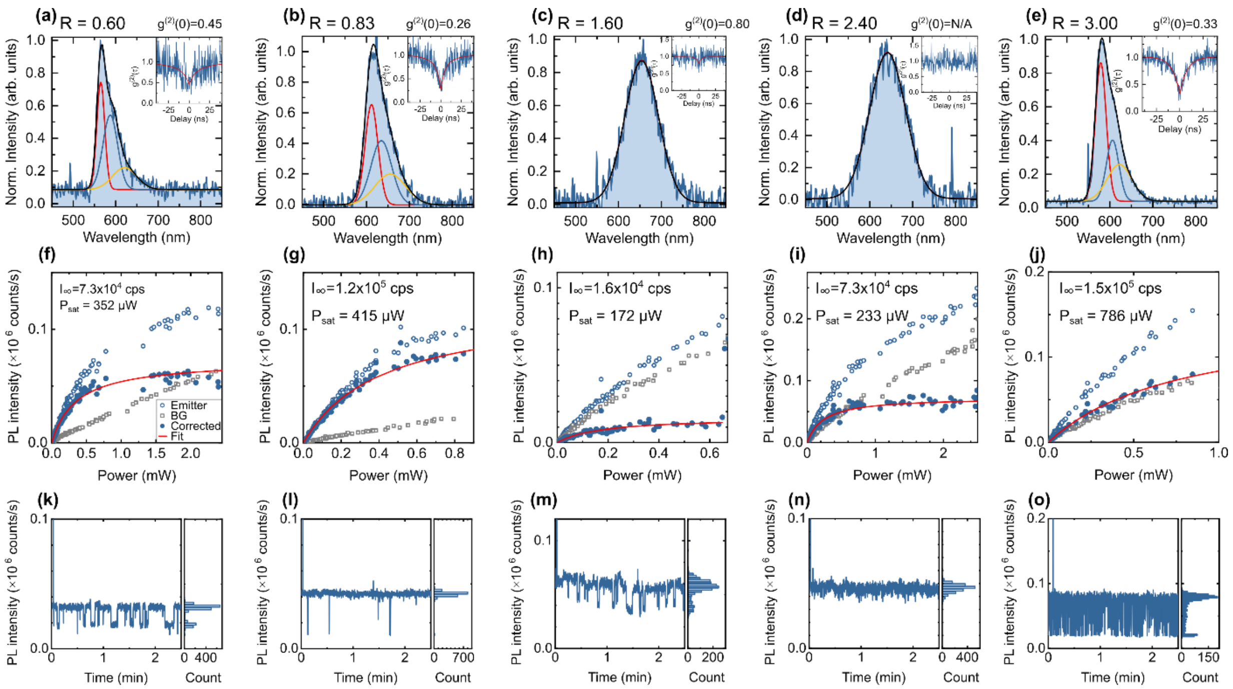

Figure S4. Photophysical properties of SPEs in N-rich PECVD SiN/Si samples after RTA shown in **Fig. S3** (red circles). All measurements were performed at room temperature. (a-e) PL spectra fitted with three Gaussian components where can be resolved. Insets: Second-order autocorrelation histograms $g^{(2)}(\tau)$ revealing single-photon emission characteristics. For emitters that do not show a clear dip at $g^{(2)}(0)$, the signal is lower than the background fluorescence. (f-j) PL saturation curves for the corresponding

emitters in (a-e), illustrating the saturation behavior typical of single-photon emitters. (k-o) Emission intensity time traces showing stability of emitters. Measurements were performed at an excitation power of 500 uW with a sampling time of 100 ms over 150 s. Left panels: Corresponding histograms of photon emission intensity distributions, illustrating transitions between high- and low-intensity states.

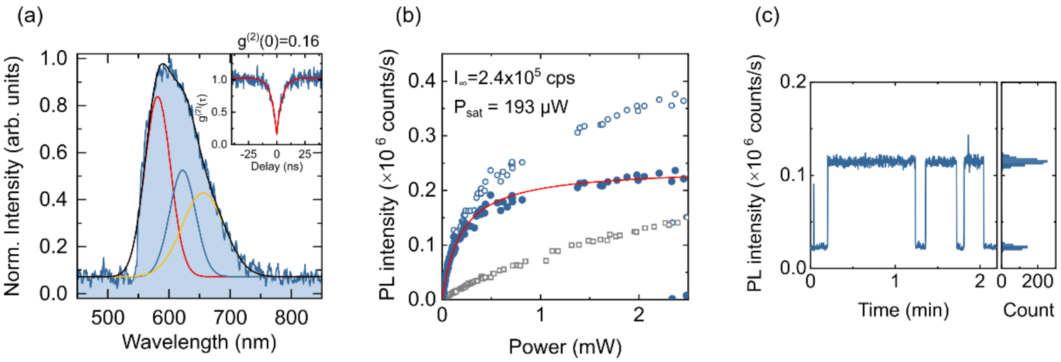

Figure S5. Photophysical properties of a representative emitter from N-rich PECVD SiN/SiO_2 sample shown in the main text (**Fig. 2d**, blue circle). (a) PL spectrum fitted with three Gaussian components. Inset: Second-order autocorrelation histogram $g^{(2)}(\tau)$, confirming single-photon emission characteristics. (b) PL intensity as a function of excitation power illustrating the saturation behavior of single-photon emission. (c) Emission intensity time trace measured at an excitation power of 500 µW with a sampling time of 100 ms over 150 s. Left panel: Corresponding histogram of photon emission intensity distributions, showing transitions between high- and low-intensity states.

Section S4. Structural and elemental composition analysis of PECVD-grown SiN samples

The cross-sectional transmission electron microscopy (TEM) specimens from the PECVD SiN samples were prepared by focused ion beam (FIB) using a Thermo Fisher Scientific Helios G4 dual beam SEM. Before FIB milling, a platinum and a carbon layer were deposited to protect the surface from ion damage. To avoid FIB induced damage, the TEM samples were ion polished using a low current at a low voltage (1 keV) multiple times as the final steps.

Scanning transmission electron microscopy (STEM) analyses and Energy-dispersive X-ray Spectroscopy (EDS) chemical mappings were carried out on a Thermo Fisher Scientific Themis Z microscope operated at 300 kV. The microscope is equipped with an electron energy monochromator, a fifth-order probe spherical aberration corrector, a high-angle annular dark field (HAADF) detector, and SuperX EDS with four silicon drift detectors. The electron probe size for STEM imaging and EDS analyses is 0.10 nm with a convergence semi-angle of 17.9 mrad. All the EDS maps were collected under the drift correction mode.

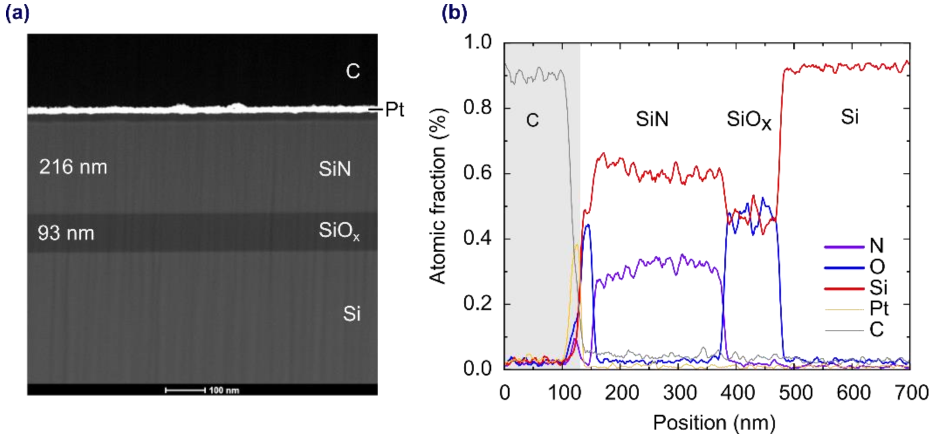

Figure S6. (a) Cross-sectional high angle annular dark field (HAADF) scanning transmission electron microscopy (STEM) micrograph of the Si-rich PECVD SiN/SiO_2 structure (R=0.1), with clear layers of SiN (216 nm) and SiO_2 (93 nm). The top layer consists of platinum and carbon, deposited to protect the sample during cross-sectional lamella preparation via focused ion beam (FIB). (b) TEM energy dispersive spectroscopy (EDS) profile showing the atomic fraction of elements across the structure, including nitrogen (N), oxygen (O), silicon (Si), platinum (Pt), and carbon (C). The EDS data provides insight into the elemental composition and distribution within the SiN, SiO_2, and Si layers.

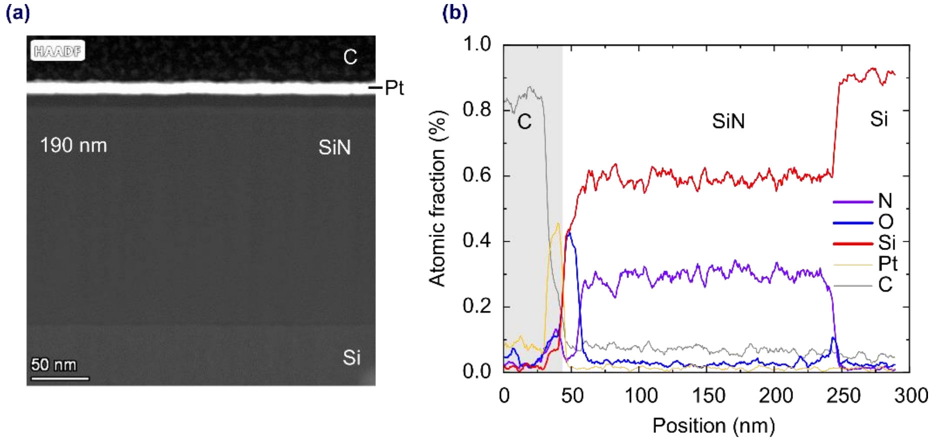

Figure S7. (a) Cross-sectional high angle annular dark field (HAADF) scanning transmission electron microscopy (STEM) micrograph of the Si-rich PECVD SiN/Si structure (R=0.1), with a clear 190-nm-thick SiN layer. The top layer consists of platinum and carbon, deposited to protect the sample during cross-sectional lamella preparation via focused ion beam (FIB). (b) TEM energy dispersive spectroscopy (EDS) profile showing the atomic fraction of elements across the structure, including nitrogen (N), oxygen (O), silicon (Si), platinum (Pt), and carbon (C). The EDS data provides insight into the elemental composition and distribution within the SiN and Si layers.

The EDS analysis of the SiN layer shows an atomic fraction of approximately 60% Si and 30% N, indicating a silicon-rich film. This deviates from the stoichiometric Si_3N_4 ratio (expected 3:4 for Si) and aligns with the precursor ratio (R=0.1) used in our growth process, where lower nitrogen content leads to excess silicon

incorporation. The same SiN composition was observed in PECVD-grown films on both Si and SiO_2-coated Si substrates, suggesting that the thick oxide layer has no significant impact on material composition, even after thermal annealing (**Fig. S6**, **Fig. S7**). The oxide layer observed on top of the SiN films results from O_2 plasma cleaning, which was performed (100W RF power for 10 minutes) to remove surface contaminants. However, this process led to the formation of an oxide layer up to 20 nm thick.

Additionally, the EDS data for the nominal SiO_2 layer (HDPCVD-grown for this sample) show a significant deviation from the expected stoichiometric composition. Instead of the ideal 1:2 atomic ratio of Si, we observe nearly equal atomic fractions for both elements (~50% Si and ~50% O), which suggests that the film may be closer to SiO (silicon monoxide) in composition. Such a deviation could be a result of the specific growth conditions or post-deposition high-temperature annealing. Further investigation is required to confirm whether this non-stoichiometric SiO formation is indeed due to the processing steps used in our work.

Section S5. Slow annealing of PECVD SiN/Si sample

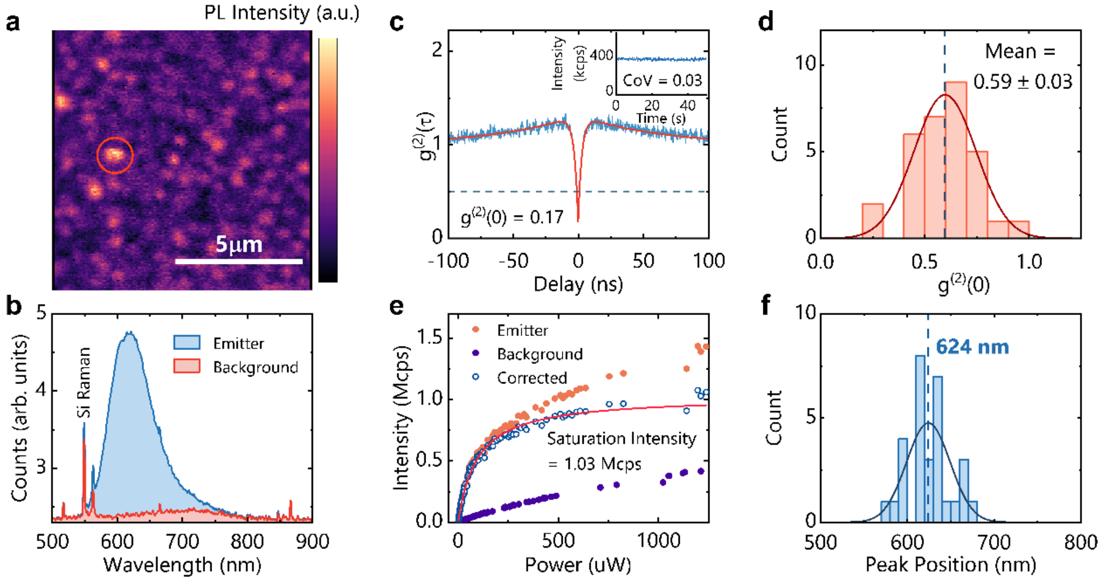

Figure S8. Photophysical properties of a Si-rich PECVD SiN/Si sample (*R*=0.1) with the SiN layer thickness of 200 nm after slow annealing treatment in conventional furnace: at 1000°C under Ar for 60 minutes, followed by an additional 60 minutes at 1100°C under N_2. (a) Confocal PL intensity map reveals a high density of color centers formed by the slow annealing process; a representative emitter circled in red is selected for further analysis. (b) PL spectra of the emitter (blue) and background (red). The emitter spectrum peaks around 625nm, which is red-shifted compared to HDPCVD SiN/SiO_2 SPEs. (c) Autocorrelation of the emitter circled in (a) with single photon purity of 83% ($g^{(2)}(0)$ = 0.17). Inset: intensity vs time measurements show high photostability with the coefficient of variation of CoV = 0.03. (d) $g^{(2)}(0)$ distribution taken from around 30 SPEs reveals a mean antibunching value of 0.59±0.03. (e) Saturation measurements taken from the emitter (orange), fluorescence background (purple) and emitter with background correction (dark blue). Fitting the background-corrected saturation shows a saturation intensity of 1.03×10^6 counts per second (cps) at ~300 μW excitation power. f) PL spectra peak position statistics; the mean peak position is 624 nm.

Here, we explored alternative thermal annealing processes beyond RTA to enhance SPE brightness and gain deeper insight into their properties. The process involved slow annealing of a Si-rich PECVD SiN/Si sample (*R*=0.1) in a horizontal furnace (ProTemp Products) at 1000°C under Ar for 60 minutes, followed by an additional 60 minutes at 1100°C under N_2. As before, confocal PL intensity maps reveal that a high density of isolated emission centers (**Fig. S8a**) are formed after the annealing process. We selected a representative emitter circled in red in **Fig. S8a**. The PL spectrum of this emitter (light blue in **Fig. S8b**) shows a broad peak centered around 625 nm which rises clearly above the PL background (red curve in **Fig. S8b**). This emitter has high single photon purity with $g^{(2)}(0)$ and photostability (**Fig. S8c**). Thus, for this particular emitter, slow annealing indeed resulted in substantial brightness improvement with good photostability. The emitter exhibited a saturation count rate of 1.03×10^6 counts per second (cps) (background-corrected saturation is shown in **Fig. S8e**), which is the highest saturation count rate observed from the PECVD SiN/Si SPEs so far. Its PL spectrum peak at 625 nm is slightly red-shifted compared to the HDPCVD SiN emitters, whose room temperature spectra are distributed around 600 nm.
